# The Association between Hypertriglyceridemia and Colorectal Cancer: A Long-Term Community Cohort Study in Taiwan

**DOI:** 10.3390/ijerph19137804

**Published:** 2022-06-25

**Authors:** Shu-Hua Hsu, De-Kai Syu, Yong-Chen Chen, Chih-Kuang Liu, Chien-An Sun, Mingchih Chen

**Affiliations:** 1 Department of Family Medicine, Fu Jen Catholic University Hospital, Fu Jen Catholic University, No. 69, Guizi Rd., Taishan Dist., New Taipei City 24352, Taiwan; crazysunnyhsu@gmail.com; 2 Graduate Institute of Business Administration, College of Management, Fu Jen Catholic University, No. 510, Zhongzheng Rd., Xinzhuang Dist., New Taipei City 242062, Taiwan; 059435@mail.fju.edu.tw; 3 Department of Orthopedics, Fu Jen Catholic University Hospital, Fu Jen Catholic University, No. 69, Guizi Rd., Taishan Dist., New Taipei City 24352, Taiwan; gladiator711124@gmail.com; 4 Master Program of Big Data in Biomedicine, College of Medicine, Fu Jen Catholic University, No. 510, Zhongzheng Rd., Xinzhuang Dist., New Taipei City 242062, Taiwan; 137159@mail.fju.edu.tw; 5 Data Science Center, College of Medicine, Fu Jen Catholic University, No. 510, Zhongzheng Rd., Xinzhuang Dist., New Taipei City 242062, Taiwan; 6 Department of Urology, Fu Jen Catholic University Hospital, Fu Jen Catholic University, No. 69, Guizi Rd., Taishan Dist., New Taipei City 24352, Taiwan; 7 Department of Public Health, College of Medicine, Fu Jen Catholic University, Xinzhuang Dist., New Taipei City 24205, Taiwan; 8 Artificial Intelligence Development Center, Fu Jen Catholic University, No. 510, Zhongzheng Rd., Xinzhuang Dist., New Taipei City 242062, Taiwan

**Keywords:** hypertriglyceridemia, colorectal cancer, diabetes mellitus

## Abstract

(1) Background: Colorectal cancer (CRC) is the third most common malignancy and the second leading cause of cancer deaths worldwide. It often diagnosed at advanced stages, and with increasing incidence at younger generation. CRC poses a heavy financial burden and a huge public health challenge nowadays. Lipoproteins and serum lipids may have an influence on carcinogenesis by making oxidative stress, inflammation, and insulin resistance. Dyslipidemia plays a potential role in the risk of CRC. The purpose of this study is to use nationally representative samples to determine epidemiologic characteristics of CRC in the Taiwanese population, and to evaluate the associations between baseline levels of lipid profile and their effect on risk of colorectal cancer (CRC) comprehensively and quantitatively. The control of dyslipidemia in primary and secondary prevention may reduce the disease burden of CRC. (2) Methods: This is a nationwide long-term community-based prospective cohort study. Data were retrieved from the nationwide population-based Taiwanese Survey on Hypertension, Hyperglycemia and Hyperlipidemia (TwSHHH). Variables were estimated by the Cox proportional hazards model which was then further adjusted for age. We also calculated the relative ratios (RRs) of CRC for joint categories of serum cholesterol, triglyceride (TG), low-density lipoproteins cholesterol (LDL-C), and high-density lipoprotein cholesterol (HDL-C) level, and to examine their combined effect and statistical interactions. (3) Results: Male, age, waist circumference, diabetes mellitus (DM), high TG, high cholesterol level, smoking history, and metabolic syndrome were proved to increase the risk of CRC. In addition, DM patients with a TG level ≥150 mg/dL and cholesterol ≥180 mg/dL had a 4.118-fold higher risk of CRC as compared with a TG level <150 mg/dL and cholesterol level <180 mg/dL, which was a significant difference (95% CI, 1.061–15.975; *p* = 0.0407). (4) Conclusions: Patients with DM should control TG and cholesterol level through diet, exercise, or taking medications more aggressively, not only for preventing cardiovascular disease, but also for first prevention of CRC. The study can be valuable for the clinicians and policy makers to implement more precisely goals about dyslipidemia management.

## 1. Introduction

Colorectal cancer (CRC) is the second most common cause of cancer death worldwide and the third leading cancer diagnosed in the United States [1]. There are an estimated 1.93 million new CRC cases diagnosed, and 0.94 million CRC-caused deaths in 2020 worldwide. The global new CRC cases are predicted to reach 3.2 million in 2040 [2,3]. In Taiwan between 2013 and 2016, CRC was the first leading cancer for males and second in females [4]. CRC has increased incidence in younger generations, while it is often diagnosed at advanced clinical stages [5,6]. In 2017, CRC is the 36th leading cause of disease burden globally, and is the fourth leading cause of cancer burden, behind only lung cancer, liver cancer, and stomach cancer [7]. In the absence of screening, lifetime CRC-related costs of $7.286 million per 1000 50-year-olds [8]. CRC still poses a heavy financial burden and a huge public health challenge nowadays. As Taiwan has been Westernized over the past decades, the mortality rate from diseases including CRC has increased gradually for both males and females since 1971 [9]. CRC is considered primarily a “lifestyle” disease. Associated factors including age, gender, genetics, obesity, low physical activity, high animal fat diets, smoking, and alcohol consumption are considered to be potential risk factors [10]. Earlier epidemiological studies have reported a great variation in the relationship between blood lipid levels and CRC. In a large-scale cohort study in Sweden, the authors analyzed the association between serum cholesterol levels and the risks of CRC in male patients and found a positive relative risk of 1.65 among men with cholesterol levels ≥276 mg/dL [11]. Other studies have claimed that increased blood cholesterol levels elevate the risk of CRC and do not have any protective effect on its occurrence or progression [12,13,14]. This finding suggests that high blood cholesterol levels may independently increase the risk of CRC. However, in the Framingham cohort, cholesterol levels less than 190 mg/dL were associated with a significantly increased risk of CRC [15]. Furthermore, one study failed to show any association between cholesterol levels and cancer [16].

Dyslipidemia is recognized as a prominent risk factor for cardiovascular diseases such as atherosclerosis [17]. Its prevalence is gradually increasing and is a leading cause of morbidity and mortality globally. In addition, the literature presented that lipoproteins and serum lipids may influence carcinogenesis by making oxidative stress, inflammation, and insulin resistance [18,19]. An American College of Cardiology report has reported that 39% of the global population has higher cholesterol levels, and more than one-half of those individuals lived in higher-income countries [20]. From 2002 to 2010, the prevalence of dyslipidemia increased from 18.6% to 33.97% [21]. In Taiwan from 1996 to 2006, dyslipidemia adolescent prevalence significantly escalated from 13% to 22.3% [22]. In adults, the prevalence rates of hypercholesterolemia, hypertriglyceridemia, an elevated low-density lipoprotein-cholesterol (LDL-C) level, and a low high-density lipoprotein-cholesterol (HDL-C) level for men and women were 53.3% and 48.2%, 29.3%, and 13.7%, 50.7% and 37.9%, and 47.4% and 53%, respectively [23]. Dyslipidemia remains undertreated despite screening tests being readily accessible. Previous studies have reported adults with high dyslipidemia prevalence rates but low awareness and management rates [24,25]. A 2013 study in Korea has shown the prevalence of dyslipidemia was 16.58% in middle-aged adults, but the diagnosis and treatment rates are only 11.9% [26]. Statin is one kind of medication for dyslipidemia treatment; however, 10% to 30% of patients never filled out their first statin prescriptions [27]. In addition, roughly 50% of patients discontinued statin therapy within their first year, with discontinuation rates ranging as high as ≥75% at two years [28], as reported in previous literature. Since there are no symptoms or complications presented during the early stage of dyslipidemia, most patients usually do not have enough knowledge and would not treat it appropriately. However, if dyslipidemia is one of the risk factors for CRC, more aggressive dyslipidemia control is very important for the primary prevention of CRC to lower the disease burden.

Previous studies on the association between CRC and dyslipidemia were mainly focused on cholesterol, with a few studies considering the separate effects of triglyceride (TG), LDL-C, and HDL-C. There are conflicting results with a few studies have discovered an increased risk of CRC with high TG or cholesterol concentrations [29,30], other studies identifying no association or reverse effect [31,32], and only a few data of HDL and LDL associated with risk of CRC [33,34]. Further clarification and establishment should be made for a comprehensive and quantitative assessment of the association between dyslipidemia and CRC.

CRC is not only a significant problem in clinical practice but also a critical challenge for public health. To establish prevention guidelines, we aim to (1) investigate whether dyslipidemia is a risk factor of CRC, (2) evaluate the associations between baseline levels of cholesterol, TG, LDL-C, HDL-C, and (3) to analyze their combined effect on the risk of CRC. The results of this study can be valuable for clinicians and policymakers to implement more precisely the goal of dyslipidemia management. The control of dyslipidemia in primary and secondary prevention may reduce the disease burden of CRC.

## 2. Materials and Methods

### 2.1. Study Population

**Figure 1:**
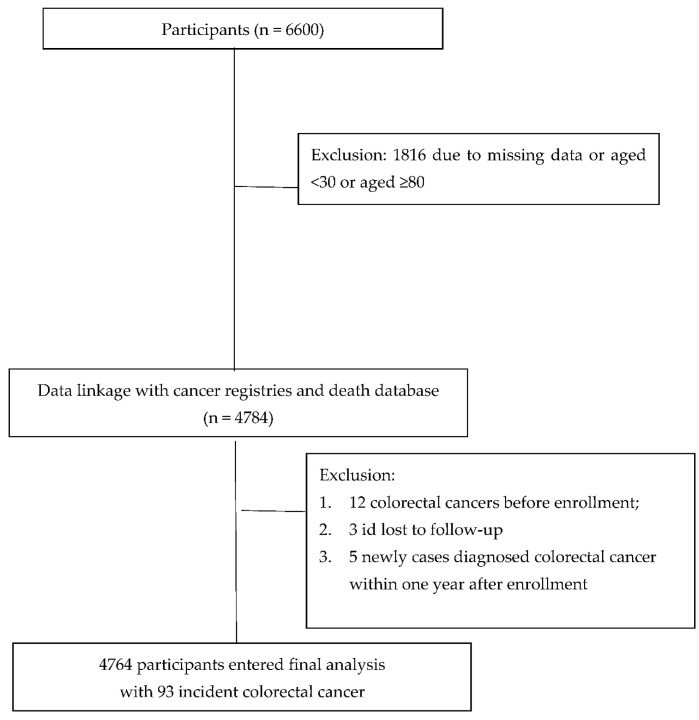
Flow of study participants in long-term follow-up study on colorectal cancer.

The data are from the Nationwide Taiwanese Survey on Hypertension, Hyperglycemia, and Hyperlipidemia (TwSHHH), conducted in 2002 [35]. The TwSHHH utilized subjects from the National Health Interview Survey (NHIS), conducted in 2001 [35] (NHIS conducted in 2001). The NHIS used a multi-stage, stratified, clustering sampling system that comprised a total of 26,685 non-institutionalized Taiwan residents from 6592 households in 1648 communities. We randomly selected 3296 households from the NHIS sampled Household Registration List in each stratum [36]. A total of 7578 (73.6% attendance rate) of 10,292 eligible subjects enrolled in the TwSHHH, but a total of 6600 adults were included in this analysis. Written informed consent was obtained from all participants in the TwSHHH. If participants had (1) missing data, and (2) age less than 30 and greater than or equal to 80 years of age, they were excluded from the first screening. Further exclusion criteria continued if participants had CRC before the enrollment, whose identification code in follow-up was lost, or either had a newly diagnosed CRC within one year after enrollment. In total, 4764 (2255 male and 2509 female) participants were analyzed in this study with 93 incident CRC, according to Figure 1. The protocols for the TwSHHH were approved by the Institutional Review Board at the Bureau of Health Promotion, Department of Health, Executive Yuan in Taiwan.

### 2.2. Data Collection

Sociodemographic characteristics, including age, sex, educational level, and menopausal status were noted during a home visit. Menopause was defined as free of menstruation for at least one year, irrespective of its causes. Anthropometrical data were measured at baseline, including body mass index (BMI), and waist circumferences (WC). BMI was expressed as body weight (kg) divided by squared body height (m^2^). WC was measured to the nearest 0.1 cm, as recommended by the World Health Organization [37]. Participants who either smoked or drank alcohol at least three times a week for at least half a year were defined and recorded as habitual smokers or drinkers.

Blood pressure was measured in a sitting position for the right arm after resting for five to ten minutes. Two readings were taken, 30 s apart. A third measurement was made if the first two differed by more than 10 mmHg. The average of the two closest readings was used. Hypertension was defined as (1) having an average systolic blood pressure (SBP) ≥ 140 mmHg; (2) having an average diastolic blood pressure (DBP) ≥ 90 mmHg; or (3) having a self-reported history of hypertension. Participants were instructed to fast for ≥8 h before blood sampling. Fasting plasma glucose (FPG) (glucose oxidase method) and TG (Bucolo method) were measured using an automated system (Vitros 550/750, Ortho-Clinical Diagnostics Inc., Johnson and Johnson Company, Rochester, NY, USA). Cholesterol, LDL-C, and HDL-C were measured using high-performance liquid chromatography (EPA-2, Helena, MT, USA). Diabetes mellitus (DM) was defined as (1) being diagnosed by a physician; or (2) being on oral anti-diabetic agents or insulin treatment; or (3) having FPG ≥126 mg/dL. Metabolic syndrome was defined by Adult Treatment Panel III criteria modified for Asians [38] as at least three of the risk factors: (1) WC measurement of ≥80 cm for women or ≥90 for men (2) TG ≥150 mg/dL (3) HDL-C <50 mg/dL for women or <40 mg/dL for men, (4) blood pressure ≥130/85 mmHg, and/or (5) fasting blood sugar ≥100 mg/dL.

### 2.3. Statistical Analysis

Person-years of follow-up for each subject were determined from the date of enrollment to the date of the diagnosis of newly developed CRC, the date of death, or on 31 December 2018, whichever was first. Incidence rates of CRC were calculated by dividing the number of incident cases by the number of person-years of follow-up. For analysis, age were classified into three groups: ≤45 years, 46–54 years, and ≥55 years; serum cholesterol levels were classified into two groups: <180 mg/dL and ≥180 mg/dL; TG classified into two groups: <150 mg/dL and ≥150 mg/dL; LDL-C classified into three groups: <110 mg/dL, 110–129 mg/dL, and ≥130 mg/dL; HDL-C classified into two groups: Male > 40 mg/dL and Female > 50 mg/dL, Male ≤ 40 mg/dL and Female ≤ 50 mg/dL; BMI into three groups: <24 kg/m^2^, 24–27 kg/m^2^, and >27 kg/m^2^, according to definition that overweight and obesity are defined as BMI ≥24 and ≥27 kg/m^2^ in Taiwan, respectively [39]. WC is divided into three groups: ≤85 cm, 86–95 cm, and >95 cm, based on median, 95%, and 99% quantile in our database. Relative risks (RRs) and 95% confidence intervals (95% CIs) for age, gender, serum cholesterol, TG, LDL-C, HDL-C level, BMI, and WC were estimated by the Cox proportional hazards model. The following analyses were adjusted for age. We also calculated the RRs of CRC in patients with or without DM for joint categories of serum cholesterol and TG level. All statistical analyses were performed using the SAS statistical package (version 9.4; SAS Institute, Inc., Cary, NC, USA). *p*-values for the trends were evaluated by the two-sided test considering 0.05 as statistically significant.

## 3. Results

Table 1 shows the basic characteristics of the participants. There were 2255 male (47.33%) and 2509 female (52.67%) in this study. The mean age was 49.37 years. Most were ≤45 years old adults (43.32%) and had an elementary school educational level (27.97%) or senior high school educational level (27.18%).

Table 2 showed the risk factors associated with CRC incidence. The univariate analysis results showed that male (compared to female, crude HR = 2.119; 95% CI: 1.386–3.241), with DM (crude HR = 3.653; 95% CI: 2.264–5.895), TG ≥ 150 (compared with TG < 150, crude HR = 2.047; 95% CI: 1.206–3.474), cholesterol ≥ 180 (compared with cholesterol < 180, crude HR = 1.723; 95% CI: 1.111–2.671), smoking history (crude HR = 1.965; 95% CI: 1.303–2.963), menopause (crude HR = 2.536; 95% CI: 1.239–5.188) and with metabolic syndrome (crude HR = 1.590; 95% CI: 1.016–2.488) were statistically significantly associated with CRC. The age of 46 to 54 and ≥55 were at a 2.677- and 5.53-fold risk of CRC compared with age ≤45. WC of 86 to 95 cm and >95 cm was at a 1.585- and 2.120-fold risk of CRC compared with a WC ≤ 85. After adjusting with age, male (aHR = 2.071; 95% CI: 1.354–3.168, *p* = 0.0008), with DM (aHR = 2.462; 95% CI: 1.507–4.024, *p* = 0.0003), TG ≥ 150 (compared with <150, aHR = 1.716; 95% CI: 1.009–2.920, *p* = 0.0463) and smoking history (aHR = 2.053; 95% CI: 1.361–3.095, *p* = 0.0006) were still statistically significantly associated with CRC.

**Figure 2:**
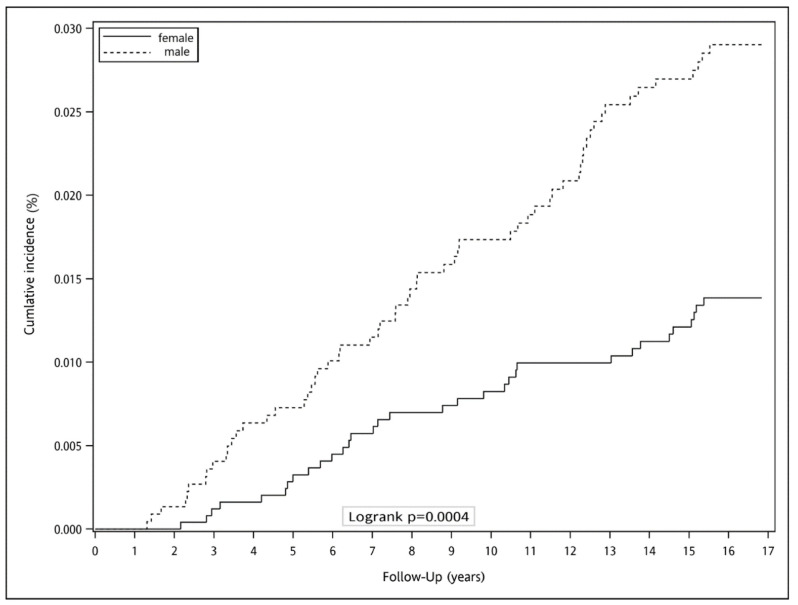
Cumulative incidence of colorectal cancer based on gender.

Figure 2 is the cumulative incidence of colorectal cancer based on gender in Taiwan. For males, the median follow-up period was 16.71 years. During the observation period, the Nelson–Aalen estimate of the cumulative incidence of male CRC was 0.00% in one year, 0.0073% in five years, 0.0173% in ten years, and 0.027% in fifteen years. For females, the median follow-up period was 16.73 years. The cumulative incidence of female CRC was 0.00% in one year, 0.0033% in five years, 0.0083% in ten years, and 0.0121% in fifteen years. The cumulative incidence of the male was significantly higher than that of the female (log-rank test *p* < 0.001; Figure 2).

Table 3 shows the association between the development of CRC in DM status and cholesterol levels and TG. In this analysis, we separated the participants into DM and non-DM groups, and classified cholesterol levels into two groups based on a cutoff value of 180 mg/dL and TG levels into two groups based on a cutoff value of 150 mg/dL, which was identified as shown in Table 2. We found that, in the DM group, those with a TG level ≥150 mg/dL and cholesterol level ≥180 mg/dL had a 4.118-fold higher risk of CRC as compared with a TG level <150 mg/dL and cholesterol level <180 mg/dL, which was a significant difference (95% CI, 1.061–15.975; *p* = 0.0407).

## 4. Discussion

This is a prospective cohort study based on a population of aged 30–80 years old in Taiwan. We have found that, in the DM group, TG ≥ 150 mg/dL and cholesterol levels ≥ 180 mg/dL caused a 4.118-fold increased risk of CRC as compared with TG < 150 mg/dL and cholesterol levels < 180 mg/dL, which reached statistical significance. To the best of our knowledge, this study is a long-term community-based cohort study with a nationally representative sample on the association between CRC risk and serum lipid profile. The accuracy collected from the national cancer registry database guaranteed the complete identification of incident cases of CRC. We determined epidemiologic characteristics of CRC in the Taiwanese population, including the fact that, for a DM patient, more active cholesterol and TG control were necessary for CRC prevention.

Despite the numerous studies that explored the relationship between blood lipids and CRC, they are still controversial. Several studies in the early 1980s reported an inverse association between cancer and serum cholesterol levels, but the association disappeared when excluding cases diagnosed within two years of enrollment [40,41,42,43]. Therefore, some researchers have suggested that lower serum cholesterol levels may reflect a response to early, undiagnosed cancers [15]. Conversely, a study based on incidence data from the Sweden nationwide cancer register presented an association between serum cholesterol levels and positive colorectal cancer. With a relative risk of 1.65 among those with cholesterol levels ≥276 mg/dL, this was statistically significant for CRC in males [11]. In the Swedish cohort, males with serum cholesterol levels <190 mg/dL did not have a higher risk for CRC [11].

Our findings revealed that, in the univariate analysis, cholesterol level ≥180 mg/dL had a 1.723-fold risk of CRC compared to cholesterol <180, which was consistent with several epidemiological works of literature previously [11,12,44,45]. A case-cohort Italian study revealed that elevated serum cholesterol is a risk factor for CRC mainly in men and postmenopausal women [29]. The evidence supporting that high cholesterol levels increase the risk for CRC partly comes from research on colorectal adenoma, which is well documented as the precursor lesion of cancer. A Korean study showed that the risk of adenomatous polyp increases with a rise in serum cholesterol levels. They compared participants with low cholesterol concentration (<199 mg/dL) with those with intermediate (200–249 mg/dL) and high concentrations (>250 mg/dL) and concluded that the groups had adjusted odds ratios of 1.82 and 2.44, respectively [13]. Another study further showed that higher cholesterol levels increased the likelihood of having colorectal villous adenoma [46], the type of adenoma at the highest risk for cancer development.

Statins remain the first-line treatment to manage hypercholesterolemia, and some studies revealed that they may show a protective effect on CRC as they can inhibit the cell cycle and induce apoptosis [47,48]. For hypercholesterolemia patients, taking statins may lower the risk of CRC. To our study, the overall relative risk of CRC may be underestimated.

Cholesterol in colorectal carcinogenesis may be involved in its effect on inflammation which may promote inhibit apoptosis and cellular proliferation [49,50]. Hypercholesterolemia is associated with oxidative stress and may play a part in cancer development [51], perhaps by altering gene expression. From the genetic perspective, the adenomatous polyposis coli (APC) gene is well recognized in regulating cellular proliferation, and its mutation plays a crucial role in initiating normal epithelium-adenomatous polyp-malignant neoplasm transformation [52]. Peroxisome proliferator-activated receptor (PPAR) is important for lipid storage and adipocyte differentiation and also is highly conveyed in the colonic epithelium [53]. In an animal model of colorectal tumors and human familial adenomatous polyposis, referred to as APC-deficient mice, Niho and their colleagues found concomitant suppression of hypercholesterolemia and intestinal polyp formation by PPARγ ligands [54]. The evidence implicates that PPARγ and the APC gene might be involved in the link between blood cholesterol and CRC, but further research on the complex genetic networks of carcinogenesis is needed to explain the widely diverse trends in CRC risk among different populations.

In our study, TG ≥ 150 mg/dL had a 2.047-, and 1.716-fold risk of CRC compared to TG < 150 separately, with statistical significance under univariate analysis and after adjusting age. The effect of TG on CRC is also conflicting in previous studies. Some studies showed no association [55,56]. However, there is an increasing amount of data supporting a positive association between hypertriglyceridemia and the incidence of CRC. Borena and their colleagues discovered that RR for the top quintile compared to the bottom quintile TG of CRC were 1.16 (95% CI, 1.06–1.26) in men and 1.15 (1.05–1.27) in women [57]. Stocks and their colleagues presented that TG was significantly associated with CRC in men (RR, 1.17; 95% CI, 1.06–1.28) [58]. According to an Austria cohort study, higher TG concentrations were associated with an increased risk of CRC both in men and women (HR, 1.56; 95% CI, 1.00–2.44) [59].

Through inducing inflammation or energy supply to neoplastic cells, serum TG concentrations may be connected to CRC risk [60]. A few studies have presented that hypertriglyceridemia is associated with frequent infections and inflammation [61,62]. From the energy supply point of view, insulin resistance represents the most plausible link between obesity and higher TG, which may lead to increased lipolysis, adiposopathy, and release of free fatty acid (FFA) into the circulation [63,64,65]. The lipolytic pathway plays a part in the progression of CRC [66], and adipose triglyceride lipase (ATGL) was important in the rate-limiting enzymes involved in lipolysis [67]. TG metabolism is instigated by ATGL through hydrolyzing TG into FFA and diacylglycerol. ATGL-mediated lipolysis releases a large amount of FFA, which is an important adaptation to the high proliferation rates of tumor cells [68]. Yin and their colleagues presented that increased ATGL positively correlates with CRC while the knockdown of ATGL inhibits the proliferation and promotes the apoptosis of CRC cells in vitro [69].

Our study displayed that DM is an important risk factor for CRC. Many Type 2 DM patients also had hypertriglyceridemia. These mechanisms are linked to insulin resistance and hyperglycemia, which resulted in the overproduction of TG-rich lipoproteins from the liver [70]. In our study, DM patients with a TG ≥ 150 mg/dL and cholesterol level ≥180 mg/dL had a 4.118-fold higher risk of CRC as compared with a TG level <150 mg/dL and cholesterol level <180 mg/dL and with significant difference (95% CI, 1.061–15.975; *p* = 0.0407). Therefore, patients with DM should control TG and cholesterol levels through diet, exercise, or taking medications more aggressively, or the risk of CRC will increase.

This study comes with strengths and limitations. The main advantage of the study is that this is a prospective cohort design. This avoided inverse causation bias, which was unlikely to occur, with nationally representative samples and with a sufficient follow-up period. We determined the epidemiologic characteristics of CRC in the Taiwanese population. Moreover, all biochemical data including lipid biomarkers were measured using the standardized and validated blood biochemistry methods with strict quality controls in a single central laboratory, thereby minimizing any measurement errors. However, since this cohort included only Taiwanese, some studies showed that there may be ethnic differences in the etiology and biology of CRC between Asians and non-Asians. Therefore, the generalization of the study findings to other ethnicities should be reconfirmed. It is hard to evaluate newly detected DM or dyslipidemia that could change during the follow-up period. Therefore, we might have underestimated the relative risk of CRC associated with DM and dyslipidemia. Some potential confounders such as medication use and family history were not measured.

## 5. Conclusions

This is an important finding for CRC prevention, especially for DM and dyslipidemia patients. It has been confirmed that the combined effect of dyslipidemia and DM will lead to an increased risk of CRC. From the point of view of preventive medications, maintaining a stable weight and blood cholesterol, TG in the appropriate range during middle adulthood is important to prevent CRC. Both the government of Taiwan and physicians can use the findings of this study as a reference when formulating screening strategies for CRC in Taiwan and also to reduce the economic burden of CRC on the national health expenditure.

## Figures and Tables

**Table 1:** Baseline characteristics of the study participants.

Variable	Subject	(%)
Gender		
Female	2509	52.67
Male	2255	47.33
Age (Years)		
≤45	2064	43.32
46–55	1248	26.2
≥55	1452	30.48
Mean (SD)	49.37	12.67
Educational level		
Elementary	1330	27.97
Illiterate	369	7.76
Junior high school	774	16.28
Senior high school	1322	27.8
College	960	20.19
Missing	9	

**Table 2:** Risk factors associated with colon cancer incidence.

Variables	Subjects	Cases	Person-Years	Incidence Rate	Crude HR (95% CI)		*p*-Value	AHR ^a^ (95% CI)			*p*-Value
**Total**	4764	93	73,812.20	126							
**Gender**											
Female	2509	33	39,702.86	83.12	1						
Male	2255	60	34,109.34	175.9	2.119	(1.386, 3.241)	0.0005	2.071	1.354	3.168	0.0008
**Age (year)**											
≤45	2064	16	33,877.66	47.23	1						
46–54	1248	25	19,812.68	126.18	2.677	(1.429, 5.013)	0.0021				
≥55	1452	52	20,121.85	258.43	5.53	(3.157, 9.689)	<0.0001				
**Educational level**											
Elementary	1330	37	19,555.84	189.2	1						
Illiterate	369	7	5094.56	137.4	0.729	(0.325, 1.635)	0.443	0.497	0.220	1.123	0.0927
Junior high school	774	21	12,179.35	172.42	0.907	(0.531, 1.55)	0.7224	1.548	0.891	2.691	0.1213
Senior high school	1322	14	21,285.94	65.77	0.346	(0.187, 0.64)	0.0007	0.712	0.373	1.357	0.3016
College	960	14	15,546.27	90.05	0.473	(0.256, 0.876)	0.0172	0.928	0.489	1.762	0.8192
**Diabetes Mellitus**											
Never	4323	71	67,921.49	104.53	1						
Have	437	22	5823.87	377.76	3.653	(2.264, 5.895)	<0.0001	2.462	1.507	4.024	0.0003
**Hypertension**											
Never	3568	64	56,814.56	112.65	1						
Have	1194	29	16,964.07	170.95	1.528	(0.985, 2.37)	0.0584	0.883	0.556	1.402	0.5966
**Cholesterol (mg/dL)**											
<180	2090	29	32,365.95	89.6	1						
≥180	2672	64	41,412.84	154.54	1.723	(1.111, 2.671)	0.0151	1.396	0.898	2.172	0.1386
**LDL (mg/dL)**											
<110	1791	26	27,879.83	93.26	1						
110–129	1433	32	22,161.75	144.39	1.547	(0.922, 2.596)	0.0983	1.307	0.777	2.199	0.3125
≥130	1538	35	23,737.21	147.45	1.58	(0.951, 2.625)	0.0772	1.156	0.691	1.933	0.5816
**TG (mg/dL)**											
<150	3294	58	51,470.89	112.69	1						
≥150	506	18	7798.17	230.82	2.047	(1.206, 3.474)	0.0079	1.716	1.009	2.920	0.0463
**HDL (mg/dL)**											
Male >40 Female >50	3491	68	54,569.16	124.61	1						
Male ≤ 40 Female ≤50	1271	25	19,209.63	130.14	1.048	(0.662, 1.657)	0.8422	1.077	0.681	1.703	0.7515
**Body Mass Index (kg/** **m^2^)**											
<24	2441	38	38,528.66	98.63	1						
24–27	1239	26	19,153.57	135.74	1.376	(0.836, 2.267)	0.2093	1.220	0.740	2.011	0.4356
>27	628	17	9802.24	173.43	1.757	(0.992, 3.113)	0.0534	1.596	0.900	2.828	0.1094
**Waist circumference (cm)**											
≤85	3025	48	47,717.51	100.59	1						
86–95	1286	31	19,460.29	159.30	1.585	(1.009, 2.491)	0.0455	1.215	0.768	1.923	0.4047
>95	446	14	6577.19	212.86	2.120	(1.169, 3.845)	0.0134	1.645	0.901	3.001	0.1049
**Smoking habit**											
Never	3383	53	53,204.58	99.62	1						
Ever	1374	40	20,490.81	195.21	1.965	(1.303, 2.963)	0.0013	2.053	1.361	3.095	0.0006
**Drinking habits**											
Never	3426	65	52,972.80	122.7	1						
Ever	1331	28	20,722.59	135.12	1.1	(0.706, 1.713)	0.6742	1.295	0.828	2.024	0.2575
**Menopause**											
Never	1454	12	23,865.14	50.28	1						
Ever	1053	20	15,814.44	126.47	2.536	(1.239, 5.188	0.0108	1.028	0.324	3.260	0.9627
**Metabolic syndrome**											
Never	3683	66	58,031.27	113.73	1						
Have	1027	27	14,989.93	180.12	1.590	(1.016, 2.488)	0.0424	1.139	0.720	1.800	0.5786
**Exercise**											
Never	3635	66	56,283.23	117.26	1						
Have	1127	27	17,495.07	154.33	1.316	(0.841, 2.059)	0.2298	1.017	0.647	1.598	0.9432
**Animal fat diet**											
Low	2758	50	42,901.11	116.55	1						
High	2005	43	30,894.37	139.18	1.195	(0.795, 1.796)	0.3921	1.168	0.777	1.757	0.4541

Abbreviations: LDL, low-density lipoprotein; TG, Triglyceride; HDL, high-density lipoprotein; ^a^: Adjusted with age Cox proportional hazards regression.

**Table 3:** Combination effects for cholesterol, triglyceride (TG) level, and colorectal cancer risk in DM and non-DM group.

	DM		Non-DM	
	TG < 150 mg/dL	TG ≥ 150 mg/dL	TG < 150 mg/dL	TG ≥ 150 mg/dL
Cholesterol				
<180 mg/dL	1.00	1.326	1.00	0.800
	(Referent)	(0.137–12.820)	(Referent)	(0.186–3.449)
≥180 mg/dL	1.531	4.118 *	1.339	1.761
	(0.381–6.160)	(1.061–15.975)	(0.751–2.388)	(0.768–4.035)

Adjusted for age, gender, diabetes mellitus comorbidity, triglyceride level, and total cholesterol level of colorectal cancer. * *p* = 0.005.

## Data Availability

Data are available from the Health and Welfare Data Science Center, HWDC. Due to legal restrictions imposed by the government of Taiwan in relation to the “Personal Information Protection Act”, the data cannot be made publicly available. Requests for data can be sent as a formal proposal to the https://dep.mohw.gov.tw/DOS/lp-2503-113-3-20.html.
